# Factors influencing refusing of flu vaccination among pregnant women in Italy: Healthcare workers’ role

**DOI:** 10.1111/irv.12600

**Published:** 2019-01-08

**Authors:** Emilia Prospero, Sara Galmozzi, Valentina Paris, Gessica Felici, Pamela Barbadoro, Antonella D'Alleva, Gemma Zocco, Andrea Ciavattini

**Affiliations:** ^1^ Department of Biomedical Sciences Section of Hygiene Università Politecnica delle Marche Ancona Italy; ^2^ Woman's Health Sciences Department Gynecologic Section AOU Ospedali Riuniti Ancona Ancona Italy

**Keywords:** flu vaccination, healthcare workers, hesitancy, pregnant women

## Abstract

**Background:**

Pregnant women are at increased risk of influenza complications. Influenza vaccine provides them a substantial protection.

**Objective:**

The aim of this study was to investigate determinants associated with non‐adherence to influenza vaccine recommendations in pregnant women in Italy.

**Methods:**

A cross‐sectional study has been carried out among pregnant women attending their follow‐up visit in some mother and child services in a Region of Italy from October 2016 to January 2017. The study protocol was approved by the local research Ethics. A self‐administered close‐ended questionnaire has been administered to the pregnant women. Differences in background, socio‐demographic characteristics, knowledge and attitudes towards flu vaccine were tested in vaccinated and unvaccinated women. Multivariate analysis was performed to control for confounding factors.

**Results:**

Three hundred and sixty‐six women answered the survey (97% response rate) and 96.1% (348) declared of being unvaccinated against influenza during the 2016‐2017 influenza season. Frequent reasons for refusing vaccination were drugs objection and concerns about vaccines’ effects. According to the refusal attitude, influenza knowledge was low in the group. Moreover, analysis showed that low adherence to vaccination is associated to lacking promotion of vaccination to pregnant women carried out by healthcare workers (*P* < 0.005).

**Conclusions:**

Healthcare workers have a key role in assisting women during the gestational period, so their active involvement in vaccination promotion is essential. It is necessary to improve health care workers’ knowledge about vaccine relevance in protecting pregnancy and their communication skills to properly inform pregnant women.

## 1 BACKGROUND

Influenza illness has been associated with increased rates of miscarriage, stillbirths, neonatal deaths, preterm deliveries and reduced birth weight.^1^, ^2^ Moreover, pregnant women despite others are at increased risk of influenza complications, including hospitalization and admission to intensive care units.^3^

Influenza vaccine is safe and provides substantial protection to pregnant women, unborn foetuses and infants up to 6 months following delivery.^4^, ^5^ In fact, since 2005, the World Health Organization (WHO) has recommended including immunization for pregnant women into national influenza vaccination programs, restating its recommendations in the 2012 position paper.^6^ In Italy, vaccination is offered for free to all women who, at the beginning of the epidemic season, are in the second or third trimester of gestation.^7^

Despite its clinical importance and the health policies, influenza vaccination coverage in pregnant women is low in Europe (23.7%),^8^ especially in Italy (2%).^9^, ^10^, ^11^

Wider acceptance of maternal immunization has been hampered by the perception that limited data about safety are available.^12^

The aim of this study was to investigate determinants associated with non‐adherence to influenza vaccine in pregnant women.

## 2 METHODS

A cross‐sectional study has been conducted over a 4‐month period from October 2016 to January 2017, covering the typical epidemic influenza period in Italy. The Study was conducted during flu vaccination campaign, and vaccine was made available to expectant mothers in all involved health services during period.

This survey was carried out by means of a self‐administered close‐ended questionnaire which was distributed by trained medical staff in outpatient of obstetrics and gynaecology departments. Services that accepted to participate to the investigation were five local obstetrics and gynaecology counselling facility and the two biggest hospitals in the Marches, a central Italy Region.

The study was conducted according to the Declaration of Helsinki and current legislation and approved by the local research ethics committee of Marches Region.

In selected gynaecology and obstetric units, a resident or a nurse have informed potential participants about the survey while they were waiting for their medical appointment. After the informative moment, questionnaire was distributed to collaborative pregnant women who signed informed consent.

The minimum sample size necessary for this study was 280 women, calculated with the formula *z* ^2^ pq/*d* ^2^ assuming that p (non vaccination rate) = 76% (prevalence was taken from available scientific literature^8^, ^13^, ^14^, ^15^), for confidence level of 95% and an error rate of 5%.

A convenience sampling method was used to recruit participants. Eligibility criteria for participation were as follows: age between 18 and 45 years old, English or Italian language, current (first‐third trimester)/planned pregnancy at the time of the study. The questionnaire included several items divided into three sections. The first section included socio‐demographic characteristics (age, nationality, educational level, marital status, occupation) and background information about the potential to influenza exposure in the occupational settings (such as being occupied in regular duties, temporary change of duties, evaluation of potential biological risk exposure), obstetrics data (gestational age at the time of the interview, obstetric history like miscarriage, previous pregnancy) and comorbidities (diabetes mellitus, gestational diabetes, nephropathy, pulmonary conditions, thyroid dysfunction, immune deficiency, other); use of complimentary and alternative medicine (homeopathy, acupuncture, herbal therapy) was also investigated.

The second section investigated knowledge about influenza and vaccination. The third section regarded pregnant women's opinions about active immunization (reasons to get vaccinated or not against a set of pathogens).

Face and content validity were evaluated through the review of the questionnaire's items by obstetrics and public health external experts and by literature review. The final version of questionnaire was validated by an experts’ panel that judged the revisited version as clear, simple and with no doubts. Concurrent construct validity was analysed by comparing couples of conveniently designed items within the instrument measuring the same concepts.

The questionnaire was pretested with a random sample of potential participants. The reliability coefficient for dichotomous variables (Kuder‐Richardson test) was 0.834.

Data were analysed using stata 15 (Stata‐Corp, College Station, TX, USA), differences in background and other characteristics of unvaccinated and vaccinated women during pregnancy were tested using *χ* ^2^ tests or Fisher's exact test. To remove confounding factors and to identify independent features associated with negative attitude to receive the influenza vaccine, a multivariate analysis has been performed to account for the locally clustered organization of healthcare services. A stepwise procedure was used to obtain the final model; level of significance was set at *P* < 0.05.

## 3 RESULTS

During influenza season, 377 women were recruited for this study. 97% (366) accepted to participate at the study and 96.1% (348) declared of being unvaccinated against influenza.

The mean age of participants was 32 years (SD 0.27, with 95% CI 31.78‐32.77), with a range from 18 to 45 years old.

About 42.9% (151) of women worked with Influenza high‐risk categories.

For the 43.8% (152) of women, pregnancy status was the reason for a temporary change of working duties.

Regarding women's health, 15.3% (55) expectant women had a history of chronic disease, while 6.9% (25) lived with people afflicted by chronic conditions.

The socio‐demographic and pregnancy characteristics of both groups, vaccinated and unvaccinated women, are reported in Table 1.

**Table 1:** Characteristics of pregnant women refusing flu vaccination (Chi‐square test)

Variables	Women refusing flu vaccination		Women accepting flu vaccination		*P*
	N	%	N	%	
Women	348		18		
Age (y)					
<24	23	6.61	3	16.67	0.59
25‐29	81	23.28	3	16.67	
30‐34	122	35.06	6	33.33	
35‐39	84	24.14	4	22.22	
>40	38	10.92	2	11.11	
Nationality					
Italian	305	91.04	14	77.78	0.06
Foreigner	30	8.96	4	22.22	
Education					
Middle/High school	177	51.30	9	50.0	0.91
College or above	168	48.7	9	50.0	
Marital status					
Unmarried	30	8.77	3	16.67	0.51
Married	311	90.94	15	83.33	
Divorced	1	0.29	0	0	
Job					
Housewife	45	13.12	5	27.78	0.41
Student	9	2.62	1	5.56	
Office worker	112	32.65	4	22.22	
Health care worker	19	5.54	1	5.56	
Manager	8	2.33	0	0	
Self‐employed	31	9.04	4	22.22	
Technician	25	7.29	1	5.56	
Shopkeeper	16	4.66	0	0	
Unemployed	33	9.62	1	5.56	
Other	45	13.12	1	5.56	
Pregnancy status					
Planning pregnancy	7	2.04	2	11.11	0.04
First trimester	21	6.12	1	5.56	
Second trimester	41	11.95	4	22.22	
Third trimester	274	79.88	11	61.11	
Parity					
Primiparus	214	62.57	9	56.25	0.61
Multiparus	128	37.43	7	43.75	
Miscarriage					
Yes	88	25.73	5	27.78	0.84
No	254	74.27	13	72.22	
Chronic conditions					
Yes	51	15.09	3	16.67	0.85
No	287	84.91	15	83.33	
Cohabiting with people affected by chronic conditions					
Yes	23	6.73	1	5.88	0.89
No	319	93.27	16	94.12	
Work with risk categories					
Yes	141	42.60	7	41.18	0.90
No	190	57.4	10	58.82	
Changing work					
Yes	145	44.21	7	41.18	0.80
No	183	55.79	10	58.82	
Non conventional therapy					
Yes	45	13.27	1	13.27	0.34
No	294	86.73	17	94.44	

In this section, univariate analysis (Table 1) showed no statistically significant differences between the two groups, except for the trimester of pregnancy (*P* < 0.05).

Dealing with vaccine and Influenza's knowledge (Table 2), a lower proportion of correct answer was observed in unvaccinated women than in the vaccinated ones; in fact, 51.7% (165) of unvaccinated women were not aware of vaccination safety during pregnancy and 74.7% (254) were not conscious of government recommendation on the topic.

**Table 2:** Knowledge of influenza and flu vaccination between women accepting or refusing immunization: distribution of agreement: number of “Yes” answers

Questions	Women accepting flu vaccination N (%)	Women refusing flu vaccination N (%)	*P* *
Is flu a communicable disease?	16 (88.9)	332 (90.7)	0.54
May the flu complications require hospitalization?	18 (100)	289 (73.5)	0.10
Are pregnant women at high risk for the flu complications with respect to non‐pregnant ones?	14 (87.5)	274 (74.8)	0.56
Do you know that Influenza vaccination is recommended and free for pregnant women, in Italy?	10 (58.8)	86 (23.5)	0.00
Do you think if flu vaccine is dangerous during pregnancy?	1 (5.9)	64 (17.5)	0.00
Have you been vaccinated for seasonal flu, in the last 5 y?	7 (41.2)	9 (2.4)	0.00
Did you get the flu over the past 5 y?	10 (58.8)	219 (59.8)	0.60
In the whole period of pregnancy, has a doctor, a midwife or any other healthcare worker told you about the vaccine, or how to obtain it?	6 (33.3)	16 (4.4)	0.00

* *P *<* *0.05 as significant.

In the second section, the univariate analysis showed statistically significant differences between the two groups.

Women with a negative attitude towards vaccines had lower awareness about Italian government's policies regarding vaccination during pregnancy, higher perception about flu vaccination risk, lower flu vaccination rate in the last 5 years, minor exposition to information about flu vaccination and its usefulness provided by healthcare workers (*P* < 0.05).

When women were asked to specify one or more reasons for not getting vaccinated, the motivations mentioned were as follows: drugs objection (23.13%), concerns about the effects of vaccine (18.86%), insufficient concern about the potential complications of the illness (17.79%), the perception of vaccines as a business of companies (6.41%), greater dangerousness of the vaccine compared to the virus (1.42%), low vaccine effectiveness (1.07%), absence of information about vaccine accessibility (0.71%) and husband aversion (0.36%). An important motivation that was mentioned by 65 (30.25%) unvaccinated women (on 281 respondents) was the lack of specific motivations to get immunized during pregnancy.

Using Hosmer‐Lemeshow test, factors tested in univariate's models (eg, nationality, pregnancy status, cohabitation with people who had chronic conditions, awareness about Italian government's policies about influenza vaccination, flu vaccination in the last 5 years and healthcare‐worker's promotion about vaccination) were entered into the stepwise multiple logistic regression analysis (Table 3).

**Table 3:** Multiple logistic regression about women's negative attitude to flu vaccination

Women refusing flu vaccination	Odds ratio	*P*	95% confidence interval
Nationality			
Foreigner	3.197	0.18	0.585–17.343
Pregnancy status			
First trimester	0.170	0.19	0.115–2.508
Second trimester	0.667	0.02	0.006–0.746
Third trimester	0.664	0.01	0.009–0.451
Cohabiting with people with chronic conditions			
No	0.643	0.72	0.055–7.538
Do you know that Influenza vaccination is recommended and free for pregnant women, in Italy?			
No	0.306	0.06	0.087–1.073
Have you been vaccinated for seasonal flu, in the last 5 y?			
No	0.418	0.01	0.009–0.190
In the whole period of pregnancy, has a doctor, a midwife or any other healthcare worker told you about the vaccine, or how to obtain it?			
No	0.165	0.01	0.039–0.687

The analysis showed that low adherence to vaccination is associated to a lacking vaccinations’ promotion from healthcare workers to pregnant women with OR 0.16 (95% CI: 0.039‐0.687). Moreover, having received influenza vaccination in the past, and being in the second or third trimester of pregnancy are associated to a refusal of vaccination, with OR 0.042 (95% CI: 0.009‐0.190), OR 0.67 (95% CI: 0.006‐0.746) and 0.66 (95% CI: 0.009‐0.451), respectively.

## 4 DISCUSSION

This study showed that more than 95% of the respondents did not request for the vaccination against Influenza.

As different Italian's authors showed, this high vaccination refusal is similar by the time. For example, Fabiani et al^9^ and Rizzo et al^16^ investigated influenza vaccine uptake during 2009 A/H1N1 pandemic outbreak, finding coverage rates of 2% and 4%, respectively; more recently (2017), Maurici et al^17^ and Napolitano^18^ studied immunization in pregnant women in two cross‐sectional studies conducted, respectively, in Rome and Naples: all the women recruited by Maurici were unvaccinated, while Napolitano et al found an hesitancy reaching more than 80% in their sample.

It is also important to consider comparison with others international similar studies^19^, ^20^, ^21^ which showed a high level of skepticism about Influenza vaccination, that remains a global public health problem which demands specific strategies and a particular focus on pregnant women as a high‐risk category.^19^

During the analysis, no significant differences were identified in socio‐demographic characteristics between unvaccinated and vaccinated women. It could be the effect of Italian's social and health policies that guarantee free access to healthcare services to all pregnant women, avoiding inequalities between women with different socio‐economic status and nationality.^22^

Furthermore, in consideration of National maternity protection policies, more than 40% (43.80%) of the interviewed women had to change their tasks at job, as recommended by the Italian health government.^23^This could be also related with the fact that 42.90% of the sample worked alongside with high‐risk categories.

This result is interesting because it shows that Italian pregnant women are well‐informed about general work‐related risks. Therefore, they take advantage of protection laws, but they do not have a perception of biological flu risk and consequently they do not ask for vaccination.

In our study, half of the women had a low‐medium level of education, both well‐educated and poorly‐educated people may have their own reasons to not accept immunization: while the inclination of people with lower socio‐economic status to refuse vaccination may reflect their misinformation, ignorance and perceived vulnerability, the tendency of more educated ones to avoid flu vaccination may be attributed to their overall distrust in science and suspicion of all kind of manufactured risks that are intrinsic to the modern world.^24^

Despite the limitations caused by the use of convenience sampling and close‐ended questionnaire format, it could be highlighted that the missed opportunity of vaccination for influenza in pregnant women in Italy may be associated to an under‐estimation of the risk of contracting or being harmed by influenza and lack of information. The occurrence of doubts about the need for vaccines, the fear of possible adverse events, the dissemination of misinformation, in addition to philosophical and religious beliefs, have created situations in which families and healthcare professionals have doubts about the need of vaccines.^25^, ^26^, ^27^, ^28^ Even if only 33.3% of women who declared immunization acceptance, have received information about the vaccine from health‐care workers, several authors^12^, ^28^, ^29^, ^30^, ^31^ do not agree on identifying that professionals as the main resources to implement correct knowledge on the topic.

In particular, for expectant women, different studies instead found that mothers who reported having received the influenza vaccine during pregnancy, in addition to protecting themselves and their offspring from infectious disease, were significantly more likely to complete their children vaccination.^32^, ^33^ These findings reinforce the idea to provide correct information on vaccine during pregnancy by clinicians.

The healthcare professionals’ vaccination, their knowledge about the subject and their own confidence in vaccines are essential to guide their behavior when indicating vaccines to their patients.^28^

Despite their key role, healthcare workers in Italy do not appear very compliant with recommendations of the WHO and the Italian Ministry of Health; in fact, their mean coverage of influenza vaccine among them stops at 20.8%, with rates reaching a minimum of 11.2% in younger age classes.^34^ Their low acceptance and disclosure of flu vaccination is probably the result of a combination of common distrust, lack of perceived risk and doubt about vaccine efficacy.

An appropriately vaccinated professional is more likely to prescribe vaccines, and that makes more evident the need for continuing education and training; therefore, it is important for scientific societies and medical associations to improve healthcare workers’ influenza coverage and formation about vaccine relevance in protecting pregnancy and their communication skills to properly inform pregnant women.^35^

### 4.1 Limits

This study includes a certain number of limits typical of population‐based questionnaire surveys. Firstly, the interviews were conducted in medical setting, and this probably produces a selection bias. In Italy, only a part of women resorts to public health services, hiding other factors associated to vaccination rejection. Secondly, there is a possibility of bias due to the high percentage of participants in third trimester of pregnancy. In fact, responses can be biased due to the low perception of risk for babies in the last period of gestation. Finally, the study is been conducted in a period full of general distrustful attitudes towards vaccination: Marches Region had in fact the lowest regional vaccination's coverage rate in Italy; this negative attitude could have influenced women's perception of risk‐benefits ratio.^8^

## 5 CONCLUSION

Pregnant women in Italy declined to be vaccinated due to an under‐estimation of the risk of contracting or being harmed by influenza and lack of information. To increase the acceptance of influenza vaccine, it would be necessary to improve the information offered to women by all members of the multidisciplinary team. For these reason in Italy, several scientific society, including SItI (Italian Hygiene Society), SIMPIOS (Italian Multidisciplinary Society for Infection Prevention) and SIP (Italian Pediatrics Society), have recently promoted a call to sensitize healthcare workers on the topic; as a result, the same societies subscribed, in March 2017, the Pisa Charter^36^; this document, aims to clarify vaccination worth as a prevention tool, to improve knowledge on immunization and to highlight health care workers’ role in promoting vaccination.

## CONFLICT OF INTEREST

Authors have no actual or potential conflicts of interest related to the submitted manuscript.
